# Child sexual abuse, adolescent/adult sexual violence, and sexual functioning among college women: a systematic review

**DOI:** 10.1186/s44263-024-00060-7

**Published:** 2024-05-13

**Authors:** Prachi H. Bhuptani, Elizabeth Mayer, Georgia Chan, Lindsay M. Orchowski

**Affiliations:** 1 Department of Psychiatry, Rhode Island Hospital, Providence, RI 02904 USA; 2 Department of Psychiatry and Human Behavior, Alpert Medical School of Brown University, Providence, RI USA; 3 Department of Cognitive, Linguistic & Psychological Sciences Brown University, Providence, RI USA

**Keywords:** Sexual assault, Rape, Sexual functioning, College women, Review

## Abstract

**Background:**

Sexual violence, including childhood sexual abuse and adolescent/adult sexual assault, is a major public health concern, especially for college women. Sexual violence is associated with numerous negative consequences, including difficulties relating to sexual functioning. The current systematic review aimed to synthesize the existing research literature examining the association between sexual violence on sexual functioning among college women.

**Methods:**

Only peer-reviewed articles reporting original data and written in English, which assessed for sexual functioning and sexual violence among a sample of college women, were included in the review. Articles were included if the research study assessed sexual violence occurring in childhood, adolescence, or adulthood.

**Results:**

A total of 21 articles met these inclusion criteria and were included in the synthesis of the literature. In studies of college women, sexual violence occurring in adulthood was associated with worse sexual functioning outcomes among college women in 7 of the 21 studies. Findings were mixed regarding the association between childhood sexual abuse and sexual functioning among college women. Further, in three studies, psychological symptoms (e.g., depression, anxiety) mediated the association between sexual violence in adulthood and worse sexual functioning among college women. Studies varied in what domains of sexual functioning were assessed, and as a result, a limited number of studies included assessments of the same domain of sexual functioning. Further, some studies did not assess sexual violence at multiple points in development (i.e., childhood, adolescence, adulthood).

**Conclusions:**

Future studies with longitudinal designs and a wider range of sexual functioning outcomes are needed, including studies focused on women attending 2-year and technical colleges.

**Supplementary Information:**

The online version contains supplementary material available at 10.1186/s44263-024-00060-7.

## Background

Sexual violence is a major social and public health problem [1]. Sexual violence is any nonconsensual sexual act including sexual contact, sexual coercion, facilitated sexual assault, attempted rape, and completed rape that can occur in childhood and/or adulthood [2]. Sexual violence is especially prevalent among college women in the United States [3]. For example, a recent systematic review suggested that as many as 25% of female college students in the United States may have experienced some form of sexual violence at some point in their lives [4]. Further, over half of the women in the United States experience some unwanted sexual contact in their lives, while one in four women experiences lifetime attempted or completed rape [2]. Lastly, one in four women experiences child sexual abuse (CSA) before the age of 18 [2]. Researchers suggest, however, that estimates of the prevalence of sexual violence among college women are likely to under-represent the scope of the problem [5]. Sexual violence is associated with numerous negative psychological, physical, and relational consequences [6–9]. Given the intimate nature of sexual violence, many survivors also struggle with sexual functioning following an assault [10].

When examining sexual functioning among survivors of sexual violence, comprehensive indicators of sexual functioning need to be included. Although there is no uniform definition of sexual functioning, the World Health Organization (WHO) refers to sexual functioning as sexual health and defines it as encompassing sexuality-related physical, emotional, mental, and social well-being [11]. The *Diagnostic Statistical Manual for Mental Disorder*, 5th edition ([DSM-5, [12]] refers to sexual functioning as sexual dysfunction and refers to a wide-ranging set of problems associated with an impaired ability to “respond sexually or to experience sexual pleasure.” Specific areas of sexual dysfunction described by the DSM-5 include physiological aspects such as desire, arousal, pain, orgasm, and lubrication along with psychological aspects such as satisfaction. The empirical literature has also examined sexual satisfaction as an important form of sexual function involving global sexual satisfaction and a range of specific sources such as sexual competence, sexual communication, and sexual compatibility [13]. Apart from the areas outlined by DSM-5, individuals who experience sexual violence may also experience a wider range of psychological difficulties associated with sex, such as sexual aversion [10] and negative sexual self-esteem [14, 15]. Thus, when investigating sexual functioning correlates among survivors of sexual violence, it is important to examine correlates beyond those identified by the DSM-5 such as sexual self-esteem [14–16], sexual schema [17], erotophilia [17], sex-related guilt [18], sex-related dissociation [19], sexual avoidance/aversion [10], and assertiveness [10].

Sexual functioning difficulties are widely prevalent among individuals who experience sexual violence [20]. Whereas 40–45% of women experience difficulties with sexual functioning regardless of a prior history of assault [21], approximately 60% of women who have experienced sexual abuse or assault experience some form of difficulties with sexual functioning [22, 23]. The ways in which sexual violence impacts sexual functioning is complex. For example, some studies suggest that adolescent/adult sexual assault (ASA) is associated with difficulties in some domains of sexual functioning, including lower sexual satisfaction [24] and diminished sexual desire [25], whereas CSA is related to higher levels of negative sexual self-esteem [14, 15] and lower response to sex therapy [26]. However, other studies fail to document an association between experiencing CSA or ASA and other domains of sexual functioning including sexual aversion [10], levels of sexual arousal [27], and difficulty with orgasm [27]. Given the varied impact of sexual violence on sexual functioning, it is important to synthesize prior literature on this topic.

Prior reviews have been conducted on the impact of childhood sexual abuse (CSA) on sexual functioning [20, 28–30] as well as the impact of military sexual trauma (MST) on sexual functioning [31]. Each of these reviews suggests that CSA and MST are associated with greater risk of difficulties in sexual functioning in adulthood. To date, research addressing the potential link between violence in other developmental time periods other than CSA (including violence in adolescence or adulthood) and sexual functioning has yet to be synthesized. Investigating the impact of CSA as well as sexual violence in other development time periods on sexual functioning is important given that some studies report that ASA, but not CSA, impacts overall sexual functioning [10, 27, 32], difficulties with lubrication [27], and difficulties with sexual distress [27]. For these reasons, synthesizing the literature examining the impact of CSA as well as violence at other points in the lifespan — such as adolescence or adulthood — is important when attempting to understand the impact of sexual violence on sexual functioning among college women.

The current systematic review aimed to synthesize the existing research literature examining the association between sexual violence on sexual functioning among college women. We chose to focus on both childhood and adulthood sexual violence. Several factors drove our decision to focus specifically on studies among college women. Firstly, college years are a critical period during which women explore sexual behaviors and begin to define their sexual identity [33]. Secondly, college age women report higher levels of personal distress associated with sexual problems and more concerns related to sexual functioning compared to older women [34, 35]. For example, in a study of 309 women from a Midwestern University in United States, 65.8% of college women reported a sexual dysfunction problem [25]. In reviewing the literature, we attempted to answer the following questions: (1) What is the prevalence of sexual dysfunction among college women with history of sexual violence? (2) What are the sexual functioning correlates of violence at various points in the lifespan (i.e., childhood, adolescence) among college women?

## Methods

This systematic review was conducted in accordance with Preferred Reporting Items for Systematic Reviews and Meta-Analyses (PRISMA) guidelines [36] (see Additional file 1 for PRISMA checklist). A Boolean search strategy was used to find studies in six electronic databases (EBSCO, ScienceDirect, PsycINFO, PubMed, Scopus, Web of Science). Variations of the following search terms were used for each electronic database: women, sexual trauma, sexual violence, intimate partner violence, sexual dysfunction, sexual function, and sexual well-being (see Additional file 2 for full search terms). Covidence systematic review software was used to retrieve and organize articles [37]. Researchers also performed manual searches of review articles and references. The initial search was completed in July of 2022. There was no date range for the review, and thus, all studies published before July of 2022 were included in the review.

### Inclusion criteria, abstraction, and synthesis

**Fig. 1:**
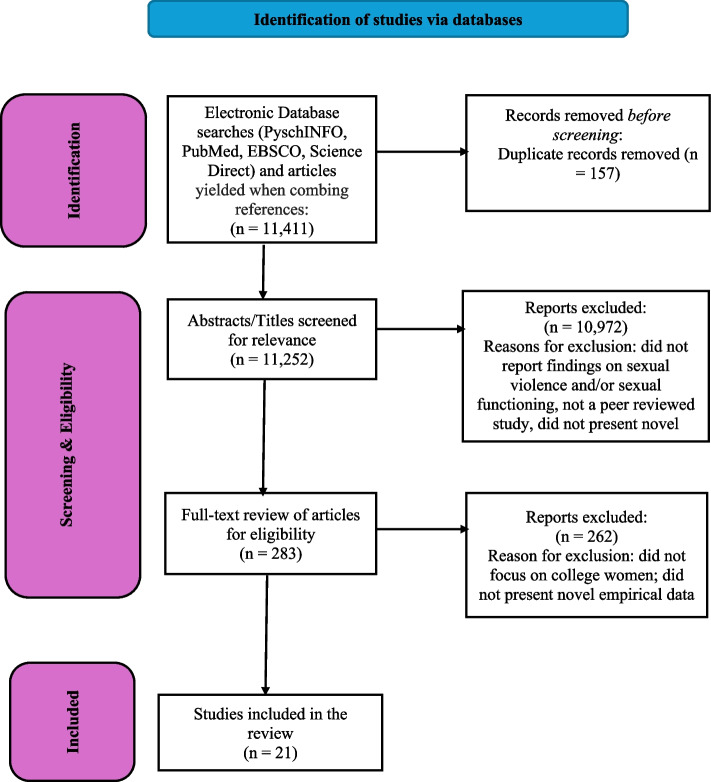
PRISMA flow diagram. Systematic identification of studies that evaluated sexual functioning in college women who experienced sexual violence. PRISMA, Preferred Reporting Items for Systematic Reviews and Meta-Analyses

Studies were included in the systematic review if they met the following inclusion criteria: (a) were a peer-reviewed article; (b) reported original data — subsamples were accepted; (c) the article was written in English; (d) the study assessed for sexual violence at some point in the lifespan; (e) the study assessed for at least one domain of sexual functioning; and (f) the study included a sample of college women. The first, second, and third authors independently screened abstracts and full-text articles. Each abstract and article was screened by at least two authors. The first and last authors performed data charting. The initial literature search resulted in 11,411 articles, 157 of which were identified as duplicates and removed. Researchers screened the title and abstract of these articles for eligibility. After initial screening, 10,972 articles were deemed ineligible for either not reporting findings on sexual violence or sexual functioning. A total of 281 articles were considered relevant for full-text screening. Of those, 262 articles were excluded for not reporting empirical data, not reporting findings on sexual functioning, or not explicitly sampling a defined cohort of college women. Dissertations were also excluded. The final 21 articles were then independently reviewed by members of the research team to assess the findings related to the impact of sexual violence on sexual functioning among college women. A PRISMA diagram illustrating the study selection process can be found in Fig. 1.

Authors used a structured and systematic data abstraction process to record data on study elements. All authors developed the abstraction form collaboratively, pilot tested the form on five articles, and deemed that no changes to the form were necessary. The process of article abstraction and comparison of data was conducted by all authors, and any discrepancies were discussed until consensus was reached. The abstraction form included inclusion and exclusion criteria, study design, sample, independent and dependent variables, assessment measures, and results. The abstraction process included results relevant to college women samples only.

## Results

Information collected on study design, measured outcomes, and evidence on the relationship between sexual violence and sexual functioning can be found in Table 1.

**Table 1:** Characteristics of studies included

Authors	Sample (age range)	Type of sexual violence	Type of sexual functioning indicator	Summary of findings
Alaxander & Lupfer (1987) [38]	*N* = 586 college women, ages 17–50 (average age: 23), did not report race or sexual identity	CSA	Sexual satisfaction, sexual functioning	• CSA is not associated with sexual functioning and sexual satisfaction
Bartoi & Kinder (1998) [32]	*N* = 175 college women, ages 18–22, 71% White, 11% Black, 10% Latinx/Hispanic, 8% “other,” 99% identified as straight	CSA, ASA	Sexual functioning, Sexual satisfaction	• ASA survivors reported higher sexual dissatisfaction and nonsexuality than those who experienced CSA or no abuse • Survivors with lifetime sexual violence were overall less satisfied with the quality of sexual relationship in comparison to women with no history of sexual abuse but did not differ on interpersonal communication with sexual partner • Neither ASA nor CSA was related to anorgasmia, sexual avoidance, sexual noncommunication, and vaginismus
Bird et al. (2017) [19]	*N* = 70 college women, ages 21–35, 70.8% White, did not report sexual identity	CSA	Sexual arousal, sex-related dissociation	• CSA severity and sex-related dissociation were positively associated — but not in the “relax and maximize” arousal condition
Fromuth (1986) [15]	*N* = 383 college women, average age:19.41 years, 98% White, did not report sexual identity	CSA	Sexual adjustment (avoidance of sexual activity, sexual desire, orgasmic capacity, having an orgasm, or anorgasmia, frequency of masturbation), sexual self-esteem	• CSA history was not associated with avoidance of sexual activity, sexual desire, sexual adjustment, self-esteem, orgasmic capacity, having an orgasm, or anorgasmia • CSA survivors engaged in higher frequency of masturbation compared to those without a history of CSA
Garneau-Fournier et al. (2017) [33]	*N* = 547 college women, ages 18–25 (94% between 18 and 20), 91% White, 98% non-Hispanic, 98% identified as straight	Lifetime sexual violence	Sexual functioning	• Sexual violence was positively associated with issues of sexual dysfunction, a greater number of sexual dysfunction problems, the likelihood of female sexual interest/arousal problems, and the likelihood of female orgasms difficulties
Halle-Ekane et al. (2021) [39]	*N* = 405, college women, ages 17–43 (average age: 21.7), did not report race or sexual identity	Lifetime sexual violence	Sexual functioning	• Women with a history of sexual violence were also about two times more likely to have female sexual dysfunction compared to those without a history of sexual violence
Jakson et al. (1990) [40]	*N* = 40 college women, ages 18–33 (average age: 23.14), did not report race or sexual identity	CSA	Sexual functioning	• 65% of survivors with history of CSA met DSM-III criteria for sexual dysfunction, with 50% reported inhibited sexual desire, 45% inhibited orgasm, 35% inhibited sexual excitement, 25% dyspareunia, and 10% vaginismus • Women with history of CSA reported lower satisfaction with sexual functioning and poorer body image, compared to those without history of CSA • Women with and without history of CSA did not differ on sexual information, attitudes, experience, drive, or fantasies
Kelley & Gidycz (2015a) [14]	*N* = 710 college women, age 18–23, 87.1% White, 5.1% Asian/Pacific Islander, 4.7% Black, 2.4% Mixed/multiracial/Other, 0.8% Latinx/Hispanic, 88.1% identified as heterosexual	CSA, ASA, revictimization	Sexual self-schema, sexual self-esteem, erotophilia	• History of CSA was associated with less control of sexual self-esteem. History of ASA was related to greater erotophilia and more positive romantic/passionate sexual self-schema but lower control, attractiveness, and moral/judgment sexual self-esteem • Revictimization was not related to any sexual functioning indicators
Kelley & Gidycz (2015b) [17]	*N* = 132 college women, ages 18–23, 85.9% White, 4.5% Mixed/multiracial, 4.4% Asian/Pacific Islander, 3.7% Black, 1.5% Latino/Hispanic, 81.5% identified as heterosexual	ASA	Sexual functioning	• Labeling of ASA as assault was indirectly associated with greater levels of sexual lubrication difficulties and sexual dissatisfaction (but not sexual desire, arousal, orgasm, and pain) via anxious coping (but not avoidance and cognitive coping)
Kelley & Gidycz (2017) [41]	*N* = 501 college women, ages 18–23, 87.7% White, 4.6% Black, 4.2% Asian/Pacific Islander, 2.2% Mixed/multiracial, 1.0% Latinx/Hispanic, 0.2% American Indian or Alaskan Native, 87.8% identified as heterosexual	CSA, ASA	Sexual functioning	• After controlling for CSA, increased anxiety and greater post-traumatic symptoms mediated the relationship between ASA and fewer sexual desire difficulties • After controlling for CSA, increased post-traumatic symptoms mediated the relation between ASA greater orgasm difficulties • After controlling for CSA, increased anxiety and depression mediated the relation between ASA and greater sexual pain • ASA and CSA were not associated with lubrication difficulties in the presence of psychological distress
Kelley & Gidycz (2019) [27]	*N* = 108 college women, average age 19.3, 87.0% White, 5.6% Mixed/multiracial, 4.6% Asian/Pacific Islander, 1.9% Black, 0.9% Latinx/Hispanic, 93.5% identified as heterosexual	CSA, ASA	Sexual functioning	• ASA severity (but not CSA severity) was associated lubrication difficulties and sexual distress • Neither ASA severity nor CSA severity were related to sexual arousal or orgasm difficulties
Kelley & Gidycz (2020) [10]	*N* = 462 college women, ages 18–23, 89.3% White, 3.7% Asian/Pacific Islander, 3.3% Mixed/multiracial, 3.0% Black, 0.4% American Indian or Alaska Native, 0.2% Middle Eastern, 97.4% identified as entirely or mostly heterosexual	CSA, ASA	Sexual aversion and assertiveness	• After controlling for CSA, lower sexual assertiveness and greater alcohol use mediated the relation between ASA and engagement in risky sexual behavior with a new partner • ASA-related post-traumatic stress symptoms did not mediate the relationship between ASA and sexual aversion • Neither ASA not CSA were associated with sexual aversion
Kilimnik et al. (2016) [24]	*N* = 126 college women, ages 18–30 (85% between 18 and 21), did not report race or sexual identity	ASA	Sexual satisfaction	• Survivors who labeled their ASA experiences as sexual assault demonstrated significantly more sexual concerns than those without history of sexual assault but did not differ from who women who did not label their ASA experiences as assault • Women with a history of ASA reported less sexual compatibility with their sexual partners and higher levels of sexual concerns (but not global sexual contentment and sexual communication satisfaction) compared to women without history of sexual assault • Depression accounted for the majority of sexual concerns between individuals with and without history of ASA
Kinzl et al. (1995) [42]	*N* = 202 college women, ages 18–30 (average age: 22), 100% White, did not report on sexual identity	CSA	Sexual functioning	• Among survivors who had experienced single incident CSA, 11.1% met DSM-IV criteria for sexual pain disorders, 11.1% met criteria for sexual desire/arousal disorder, and 27.8% met criteria for orgasm disorder • Among survivors who experienced multiple incident CSA, 15.4% met DSM-IV criteria for sexual pain disorder, 30.8% met criteria for sexual desire/arousal disorder, and 42.3% met criteria for orgasm disorder • Survivors with multiple incidents of CSA were reported sexual desire/arousal disorders significantly more than those with single-incident CSA and no history of CSA
Layh et al. (2020) [45]	*N* = 1534 college women, ages 18–25; 79.6% White, 16.5% Black, 6.2% Latinx/Hispanic, 3.1% Asian, 0.8% “other,” 95.3% identified as heterosexual	CSA, ASA	Sexual satisfaction, sexual motives	• Maladaptive sex motives (reduce their negative affect, improve their self-esteem, and obtain approval or avoid censure from their peers and sexual partners) mediated the relation between lifetime history of rape and risky sexual behavior and sexual satisfaction
Lemieux & Byers (2008) [16]	*N* = 270 college women, age 17 to 48 (average age = 23), primarily White and 96% heterosexual	CSA, ASA	Erotophobia, erotophilia, sexual self-esteem, sexual self-schema, sexual costs, sexual rewards, sexual functioning, sexual anxiety	• Women who experienced CSA with penetration were reported both a higher number and a higher level of sexual costs, more positive self-schema, greater levels of erotophilia, and lower sexual self-esteem than did the women who experienced CSA with fondling and women in no CSA group • Compared to the NO ASA Group, the ASV Group reported lower sexual self-esteem, lower sexual satisfaction, higher relative sexual costs, more sexual problems, and lower level of sexual rewards • Compared to revictimized women, the women who had experienced CSA only had fewer sexual problems and reported a lower level of sexual rewards, higher sexual costs, and lower sexual self-esteem. The women who reported ASA only reported lower level of sexual costs than the revictimized women
Meston et al. (1999) [13]	*N* = 1032 college women (*n* = 656) age 18–25, 58.1% non-Asian, 41.9% Asian, 94.3% identified as heterosexual, 3.6% bisexual, 1.2% lesbian	CSA	Sexual satisfaction	• History of CSA was associated with lower sexual drive, increased variety of sexual fantasies, and experiences, increased masturbation, increased unrestricted sexual behaviors, attitudes, and fantasies • History of CSA was not associated with body image
Orlando & Koss (1983) [43]	*N* = 116 college women, average age: 19.3, 93% White, 7% Black, did not report sexual identity	ASA	Sexual satisfaction	• Women who experience sexual contact or rape reported less sexual satisfactions compared to those who experience pressure and/or coercion
Pihgrens et al. (1993) [18]	*N* = 167 college women, ages 18 to “20 or older,” did not report race or sexual identity	CSA; ASA	Sexual functioning, sex-related guilt	• Neither ASA nor CSA were related to fantasies, sex drive, sexual satisfaction, and sex-related guilt
Rellini & Meston (2007) [44]	*N* = 699 college women, average age = 18, primarily White	CSA	Sexual functioning, sexual satisfaction	• Compared to women who did not experience CSA, women who experienced CSA endorsed higher personal distress on Sexual Satisfaction Scale • The CSA group reported greater sexual distress compared to the women who experienced nonsexual abuse, who in turn reported more distress than women who did not experience abuse. No significant group differences were observed in the sexual functioning • Vaginal penetration, fear at the time of the abuse, familial relationship with the perpetrator, and chronic frequency of the abuse were associated with sexual satisfaction but not sexual function
Turchik & Hassija (2014) [25]	*N* = 309 college women, ages 18–22, 98.7% White, 98.7% identified as heterosexual	ASA	Sexual functioning, sexual desire	• Women who reported experiencing sexual contact, sexual coercion, or rape were more likely to report a lack of sexual desire compared with those who reported no violence • Women who reported experiencing rape were more likely to report difficulty achieving orgasm compared with those who reported no violence

*CSA* Childhood sexual abuse, *ASA* Adult sexual assault, *DSM-IV Diagnostic and Statistical Manual of Mental Disorders*, fourth edition

### Study designs

Findings yielded 21 studies that have examined the impact of sexual violence experiences on sexual functioning among college women [10, 13–19, 24, 25, 27, 32, 33, 38–45]. All studies except one [19] were cross-sectional in nature, using self-report survey-based designs. The study by Bird and colleagues [19] was experimental in nature.

### Sample characteristics

Five of the 21 studies [10, 14, 17, 27, 41] examined the impact of sexual violence on sexual functioning among different subsets of the same sample of college women. Thus, the association between sexual violence and sexual functioning among female college survivors has been examined in 16 unique samples. Additionally, 2 of the 21 studies utilized samples that were recruited from college but involved both college women and community-residing women [19, 40], and did not distinguish between which women in the sample were college women or community residing women. Notably, all but one [16] studies focused on a typical 4-year university. Lemieux and Byers [16] focused on both community college and 4-year university women without examining differences between the two. No study has focused solely on community college women survivors. The earliest study was published in 1983 [43], and the latest studies were published in 2020 [10, 45]. Across studies, samples were primarly white and heterosexual (see Table 1).

### Measurement of sexual violence experiences

Seven studies focused on survivors of CSA only [13, 15, 19, 38, 40, 42, 44]. There was also variability in the definitions used to distinguish between childhood and adolescent sexual violence. For example, four studies utilized Finkelhor’s [46] definition to classify CSA, which involves experiencing sexual noncontact or contact by a perpetrator who is at least 5 years older than the survivors, was a caretaker or an authority figure, or used some form of coercion or force was used to secure the survivors’ participation [13, 15, 38, 40]. Varied age cut-off criteria were used to define CSA across these four studies. For example, two studies used 18 years and below as the age cutoff [13, 40], one used 16 years and below [15], one used 12 years and below [44], and one did not specify any age cutoff [38]. Bird and colleagues [19] used the Childhood Trauma Questionnaire-sexual abuse subscale [47] to assess CSA, without specifying any age cutoff. Kinzl and colleagues [42] used a self-designed CSA checklist, again without specifying any age cut off. Lastly, Rellini and Meston [44] used an adapted measure of CSA and defined it as activities involving genital touch, oral sex, anal penetration, or vaginal penetration before the age of 12.

Four studies focused on survivors of ASA only [17, 24, 25, 43]. Whereas three of the studies [17, 24, 43] defined ASA as unwanted sexual experiences occurring after the age of 14 and used varied versions of the Sexual Experiences Scale to measure ASA [48, 49], Turchik and Hassija [25] used Sexual Coercion Tactics Scale [50] which as the age cut-off criteria of 16 and above.

Eight studies assessed for experiences of both CSA and ASA within the study sample [10, 14, 16, 18, 27, 32, 41, 45]. All but one [16] used Finkelhor’s [46] definition to classify CSA. Lemieux and Byers [16] restricted measurement of CSA to contact behaviors (e.g., touching, fondling, intercourse) before the age of 13. Of the remaining studies, all except one [32] used the age cutoff of 14 and below. Bartoi and Kinder [32] used 16 years and below as the age cutoff for CSA. Further, all but one [32] of the studies used varied versions of the Sexual Experiences Scale to measure ASA [48, 49] with the age cutoff of 14 and above. Bartoi and Kinder [32] used the age cutoff of 16 and above to classify ASA. Lastly, two studies assessed for lifetime history of sexual violence [33, 39] and thus did not provide age cutoff.

### Indicators of sexual functioning

Across 21 studies, there was variability in the indicators of sexual functioning measured. Eleven studies examined overall sexual functioning [16–18, 25, 27, 33, 38–41, 44], seven studies examined sexual satisfaction [13, 24, 32, 38, 43–45], and three studies evaluated sexual self-esteem among survivors [14–16]. One study examined sexual schema which evaluated women’s view of themselves as sexual persons from a negative (i.e., embarrassment/conservatism) to positive (i.e., romantic, open) dimension along with erotophilia (i.e., participant’s affective response to certain sexual behaviors or stimuli) [17]. One study evaluated sex-related guilt [18]. One study measured levels of sexual arousal after watching an erotic video along with sex-related dissociation [19], whereas one study assessed sexual avoidance/aversion and assertiveness [10].

### Prevalence rates of sexual dysfunction

Only three studies provided prevalence rates of sexual dysfunction [33, 40, 42] with two using the DSM-III criteria [40, 42] and one using the DSM-IV diagnostic criteria [33]. Among women with history of CSA, 65% of survivors met DSM-III criteria for one or more sexual dysfunctions. Specifically, 50% reported inhibited sexual desire, 45% inhibited orgasm, 35% inhibited sexual excitement, 25% dyspareunia, and 10% vaginismus [40]. Another study evaluated prevalence rates of sexual dysfunction based on whether women experienced a single incident of CSA or multiple [42]. The authors found that among college women who had experienced single incident CSA, 11.1% met criteria for sexual pain disorders, 11.1% met criteria for sexual desire/arousal disorder, and 27.8% met criteria for orgasm disorder. The prevalence rates across domains increase exponentially when examining women with multiple incident CSA. Among these women, 15.4% met criteria for sexual pain disorder, 30.8% met criteria for sexual desire/arousal disorder, and 42.3% met criteria for orgasm disorder. The last study found among college women history of lifetime sexual violence, 83% (*n* = 167) experienced problems with sexual dysfunction, whereas around 44% (*n* = 167) of participants with at least one sexual dysfunction problem reported a history of lifetime sexual violence [33].

### Impact of CSA on sexual functioning

Seven studies examined the impact of CSA on sexual functioning [13, 15, 19, 38, 40, 42, 44] among adult college women and demonstrated mixed findings for psychological aspects of sexual functioning. Two studies found that women with and without history of CSA do not differ on sexual satisfaction [13, 38]. Specifically, Meston and colleagues reported that women with and without history of CSA did not differ on global sexual satisfaction and as well as specific domains such as sexual contentment, sexual competence, sexual communication, and sexual compatibility. However, two studies found women with history of CSA reported less satisfaction (overall and specific components such as decreased frequency of intercourse, poorer quality of communication, and poorer quality of orgasm) [40] and less sexual distress [44]. One study found that CSA severity was not associated with sex-related dissociation [19]. Three studies noted compared to women without history of CSA, and women with history of CSA do not differ on sexual functioning [38, 44], sexual information [40] attitudes [40] experience [40], or fantasies [40]. Along this vein, another study found that history of CSA was not associated with sexual adjustment and self-esteem [15]. In contrast, one study found that history of CSA was associated with increased variety of sexual fantasies and experiences, increased unrestricted sexual behaviors, attitudes, and fantasies [13]. Similarly, whereas one study found that women with history of CSA demonstrated poorer body image [40], another study found that history of CSA was not associated with body image [13]. Lastly, one study found that specific abuse characteristics (i.e., vaginal penetration, fear at the time of the abuse, familial relationship with the perpetrator, and chronic frequency of the abuse) were associated with sexual satisfaction but not with sexual function [44].

Studies also find mixed evidence for physical aspects of sexual functioning. Specifically, two studies noted compared to women without history of CSA, and women with history of CSA do not differ on sexual drive [40]. Similarly, one study found that history of CSA was not associated with avoidance of sexual activity, sexual desire, orgasmic capacity, having an orgasm, or anorgasmia [15]. In contrast, one study found that history of CSA was associated with lower sexual drive and increased masturbation [13]. One study found the survivors of repeated incidents of CSA significantly demonstrated more sexual dysfunction, as qualified by DSM-III, than those who experienced one incident of CSA or did not experience it all [42]. The authors also reported that individuals who did not experience CSA and those who experienced one incident of CSA did not differ on sexual functioning.

### Impact of ASA on sexual functioning

Four studies have examined the impact of ASA on sexual functioning and found consistent associations between ASA and psychological (sexual satisfaction, sexual compatibility, and sexual concerns) and physical (desire and ability to achieve orgasm) functioning [17, 24, 25, 43]. One study found that ASA severity was associated with sexual satisfaction such that women who experience sexual contact or rape reported less sexual satisfaction compared to those who experience pressure and/or coercion. One study found that women history of ASA reported less sexual compatibility with their sexual partners and higher levels of sexual concerns (but not global sexual contentment and sexual communication satisfaction) compared to women without history of SA [24]. Two studies found that ASA severity was associated with sexual functioning [25, 43]. Specifically, women who reported experiencing sexual contact, sexual coercion, or rape were more likely to report a lack of sexual desire compared with those who reported no violence [25]. In addition, women who reported experiencing rape were more likely to report difficulty achieving orgasm compared with those who reported no violence [25].

#### Impact of ASA-related labeling

Two studies found that labeling of ASA also had an impact on both psychological and physical aspects of sexual functioning [17, 24]. Specifically, one study found that labeling of ASA as assault was indirectly associated with greater levels of sexual lubrication difficulties and sexual dissatisfaction (but not sexual desire, arousal, orgasm, and pain) via anxious coping (but not avoidance and cognitive coping) [17]. Similarly, another study found that survivors who label their ASA experiences as sexual assault demonstrated significantly more sexual concerns than those without history of sexual assault [24]. However, the authors found that survivors who did not label their ASA experiences as assault were not significantly different from those labeled on sexual functioning.

#### Impact of ASA-related psychological symptoms

Four studies examined how psychological symptoms impacted physical and psychological aspects sexual functioning among ASA survivors [10, 24, 27, 41]. ASA-related intrusive symptoms were associated with orgasm difficulties and sexual distress [27]. Another study found that increased anxiety and greater post-traumatic symptoms mediated the relationship between ASA and fewer sexual difficulties, but only post-traumatic symptoms mediated the relation between ASA and greater orgasm difficulties and ASA and greater sexual pain [41]. Further, increased depression symptoms also mediated the relation between ASA and greater sexual pain. The third study found that depression accounted for the majority of sexual concerns between individuals with and without history of ASA, suggesting that depressive affectivity may explain differences in sexual concerns between these two groups [24]. The fourth study reported that ASA-related post-traumatic stress symptoms did not mediate the relationship between ASA and sexual aversion [10].

### Impact of joint experiences of CSA and ASA on sexual functioning

Eight studies assessed for both CSA and ASA within the study sample [10, 14, 16, 18, 27, 32, 41, 45].

#### Differential impact of CSA and ASA

Five studies examined the differential impact of both CSA and ASA [14, 16, 18, 27, 32]. One found that a history of CSA was uniquely related to lower control sexual self-esteem, whereas history of ASA was uniquely related to greater erotophilia and more positive romantic/passionate sexual self-schema yet lower control, attractiveness, and moral/judgment sexual self-esteem [14]. Further, one study found that women who experienced penetration during CSA reported lower sexual self-esteem than those who experienced fondling during CSA or did not experience CSA [16]. Two studies found that that ASA, and not CSA, had a detrimental impact on sexual satisfaction [16, 32]. One reported that women with history of ASA reported more sexual dissatisfaction and higher levels of nonsensuality compared to women with history of CSA and women without a history of sexual violence [32]. Further, the authors reported that women who were sexually abused in childhood did not differ on indicators of sexual functioning from women who were not victimized. Lastly, another study found that ASA severity (but not CSA severity) was associated with lubrication difficulties and sexual distress [27].

In contrast, five studies [10, 18, 27, 32, 41] find that neither ASA nor CSA were related to certain physical and psychological indicators of sexual functioning. For example, studies report that neither ASA nor CSA were related to sexual aversion [10], sexual arousal [27], orgasm difficulties [27], sexual functioning [41], anorgasmia [32], sexual avoidance [32], sexual noncommunication [32], vaginismus [32], fantasies [18], sex drive [18], sexual satisfaction [18], and sex guilt [18].

#### Additive impact of CSA and ASA

Two studies have examined additive impact of both CSA and ASA [14, 16]. The authors found experiencing both CSA and ASA was not significantly related to any sexual functioning indicators, including pain, lubrication, orgasm, desire, arousal, and overall satisfaction [14], compared to survivors who experienced only CSA or ASA. Another study found contrasting results and indicated that compared to revictimized women, the women who had experienced CSA only had fewer sexual problems and lower sexual self-esteem [16].

#### Impact of lifetime history of sexual violence

Four studies assessed lifetime history of sexual violence [32, 33, 39, 45] and largely found that lifetime history of sexual violence was related to adverse sexual functioning outcomes. One found that maladaptive sex motives (reduce their negative affect, improve their self-esteem, and obtain approval or avoid censure from their peers and sexual partners) mediated the relation between lifetime rape history and sexual satisfaction [45]. Another study reported that participants with a lifetime history of sexual violence were also about two times more likely to have difficulties with sexual functioning [39]. Another study found that women with history of lifetime sexual violence were 0.4 times likely to experience with female sexual interest/arousal problems and 0.7 times likely to experience female orgasmic problems [33]. In contrast to these three studies, one study found that women with lifetime history of sexual violence did not differ from those without a history of violence on interpersonal communication with sexual partner [32].

## Discussion

The purpose of the current review is to synthesize what is known about the relation between sexual functioning and sexual violence among college women. Specifically, the review aimed at identifying the prevalence of sexual dysfunction among college women with history of sexual violence. Additionally, results were synthesized to determine the sexual functioning correlates of *both* CSA and ASA among college women. Findings suggest that CSA is not uniformly related to varied indicators of sexual functioning among college women. For example, whereas two studies found that women with and without history of CSA do not differ on sexual satisfaction [13, 38], one study found women with history of CSA reported less sexual satisfaction [40]. Similarly, whereas one study found a history of CSA was associated with poorer body image [40], another study found that history of CSA was not associated with body image [13]. One study also report that CSA severity [27] is not associated with sexual functioning outcomes. Findings are in contrast with prior reviews on sexual functioning and CSA which note a positive association between CSA and poorer sexual functioning [20, 28, 29].

Our findings may contradict prior reviews [20, 28, 29] for several reasons. Firstly, this is the first systematic review on this question, and previous reviews were all narrative. Thus, studies that may show nonsignificant findings between CSA and sexual functioning may not be included in the prior reviews. Secondly, the review conducted by Pulverman and colleagues [20] notes that associations between CSA and sexual functioning are examined in predominantly clinical and community samples. For example, only 2 of the 12 studies exmained in this review included college sample. Thus, findings of current review in conjuction with prior review suggests that CSA may not be relevant for all indicators of sexual functioning among nonclinical college samples.

Thirdly, there is considerable variability in how sexual functioning indicators were mesaured across the studies. For example, one study examined sexual satisfaction [38], and two studies also examined body image as an indicator of sexual functioning [13, 40]. Jackson and colleagues measured global sexual satisfaction as well as satisfaction within specific such as decreased frequency of intercourse, poorer quality of communication, and poorer quality of orgasm [40]. However, Meston and colleagues [13] defined sexual satisfactions as including global sexual satisfaction and a range of specific sources of sexual dissatisfaction, including sexual contentment, sexual competence, sexual communication, and sexual compatibility [13]. Both studies found contrasting results indicating that CSA may be relevant for certain aspects of sexual satisfaction (e.g., poorer quality of orgasm) as opposed to others (e.g., sexual competence) [13, 40]. Additionally, Alexander and Lupfer [38] did not define sexual satisfaction or provide a measure description with citation which may also contribute to variability in results. Lastly, abuse characteristics (rather than the presence versus absence of CSA) may be associated with sexual functioning. For example, one study found that experiencing multiple incidents of CSA rather than one incident may lead to poorer sexual functioning [42]. Another study found that vaginal penetration, fear at the time of the abuse, familial relationship with the perpetrator, and chronic frequency of the abuse were associated with sexual satisfaction [44]. Thus, future studies investigating link between CSA and sexual functioning must examine in the role of abuse characteristics [51].

In contrast to the link between CSA and sexual functioning among college women, findings from current review suggest that both history of ASA and severity is consistently linked to varied psychological aspects of sexual functioning indicators such as less sexual compatibility with their sexual partners [24], higher levels of sexual concerns [24], and less sexual satisfaction [43]. Additionally, history of ASA is associated with physiological aspects of sexual functioning as well such as a lack of sexual desire [25] and difficulty achieving orgasm [25]. Results are in line with prior systematic review on MST, a specific and unique form of ASA, and sexual functioning [31] that found a consistent relation between MST and sexual functioning among women veteran samples. The differential impact of ASA and CSA on sexual functioning among college women is reported by two studies [27, 32]. The two studies found that ASA severity (but not CSA severity) was associated lubrication difficulties and sexual distress [27], and history of ASA (but not CSA) was associated with greater reported sexual dissatisfaction and higher levels of nonsensuality [32]. Thus, findings suggest that experiences of ASA must be attended to especially when working with college women with problems related to sexual functioning. Additionally, different mechanisms and thus different interventions may be salient in the association between ASA and physical aspects sexual functioning as well as ASA and psychological aspects of sexual functioning, a topic of future inquiry.

Review findings also suggest that the relation between ASA and sexual functioning is complex and nuanced. As a matter of fact, anxious coping [17], ASA-related intrusive symptoms [27], anxiety [41], and depressive affectivity [24, 41] mediate the relation between ASA and sexual functioning indicators. In addition to being common post-assault experiences [8], depression and anxiety share a bidirectional relation with sexual functioning such that increased depression/anxiety is associated with poorer sexual functioning and vice versa [52, 53]. Findings suggest that ASA experiences on their own may not contribute to poorer sexual functioning but rather psychological distress following an experience of ASA may contribute to poorer sexual functioning. Findings have specific implications for research and practice. Firstly, empirical investigation of sexual functioning and sexual violence must also investigate the impact of psychological distress on sexual functioning. Secondly, clinicians who work with clients who have a history of sexual violence and psychological distress must attend to challenges with sexual functioning. Lastly, although not investigated in the studies included in the review, gender norms may also play a salient role in the relation between sexual violence and sexual functioning. For example, within the US context, heterosexual sexual scripts largely guide sexual activity and assume that women will conform to feminine gender norms (e.g., passivity) and men will conform to masculine gender norms (e.g., assertiveness), and great gender conformity is associated with lower sexual satisfaction for women [54, 55]. The very act of sexual violence may reinforce these gender scripts leading to lower sexual functioning. Thus, future studies should examine the role of gender norms in the relation between sexual violence and sexual functioning.

Only three studies have provided prevalence of female sexual dysfunction reported by college women with history of sexual violence [33, 40, 42]. Two studies found that among college women with history of CSA, 11–65% of survivors met DSM-III criteria for one or more sexual dysfunctions [40, 42], whereas another study among college women with lifetime history of sexual violence found that 83% of survivors experienced problems with sexual dysfunction [33]. The prevalence rate of sexual dysfunction is higher than prevalence of female sexual dysfunction reported by college women in general (35–42%) [56, 57] and community women (12–50%) [58–60]. Thus, findings highlight the detrimental impact of sexual violence on sexual functioning. However, additional future research is required to determine the prevalence of sexual dysfunction among college women survivors. Additionally, future studies should consider using diagnostic clinical interviews for female sexual dysfunction disorders and include all relevant diagnostic criteria to improve the accuracy and specificity of prevalence of sexual dysfunction in this overlooked population.

Findings also suggest the importance of examining varied indicators of sexual functioning, beyond those defined by the DSM-5 as results highlight that experiences of sexual violence may be associated with certain sexual functioning indicators that are not captured by the DSM-5 criteria for sexual dysfunction [e.g., sexual compatibility [24] or sex-related dissociation [19]]. Additionally, there is need for uniformity in measurement of similar domains of sexual functioning. Specifically, there is difference in conceptualization of similar domains of sexual functioning within studies evaluating similar outcomes such as sexual functioning [17, 18, 25, 27, 33, 38–41] or sexual satisfaction [13, 24, 32, 38, 43, 45], However, varied conceptualization may contribute to inconsistent findings noted in the review. Developing uniformity in measurement is recommended to improve understanding of the link between sexual functioning and sexual functioning.

All studies evaluated in this review are using the typical 4-year university samples, which overlooks community college women. Sexual violence is a pervasive concern among community college women as well with research estimating that 25% of women experience sexual violence prior to entering community college [61] and 12.7% of women experience sexual violence while enrolled in community college [62]. Further, compared to students enrolled at 4-year colleges, women at community colleges display particularly higher rates of sexual risk behaviors and greater physical and mental health concerns [63–65]. Thus, it is imperative that the outcomes of sexual violence, particularly sexual functioning, must be evaluated among college community samples as well. It should also be noted that two of the studies included in the review [19, 40] include both college women and women residing in the community, but did not distinguish between these two groups in the study sample. Future research should take care to ensure that groups are clearly delineated, so that outcomes that might be utilized to inform practice, intervention and prevention activities in college health centers can be readily delineated from the research.

Relatedly, samples in studies identified in the review were predominantly White and heterosexual which limits investigation into how race and sexual orientation may impact relation between sexual violence and sexual functioning. Racial and sexual minority women experience sexual violence at disproportionately higher rates compared to White and heterosexual women [66]. Racial and sexual minority individuals also rate higher or lower on varied aspects of sexual functioning. For example, African-American women reported greater levels of sexual satisfaction compared to White women [67]. Another study found that Black women tend experience lower desire and decreased pleasure compared to White women, whereas White women report experiencing more sexual pain compared to Black women [60]. Similarly, women report differently on sexual functioning based on their sexual identity [68]. For example, a meta-analysis demonstrated that lesbian women experience more orgasms than heterosexual women [69]. Given these important racial and sexual differences in prevalence of sexual violence and sexual functioning, future studies that oversample for racial and sexual minority are needed to investigate how race or sexual identity may impact the relation between sexual violence and sexual functioning.

Notably, none of the studies examining ASA and sexual functioning distinguished between assault experienced before college and during college [17, 24, 25, 43]. Studies used the cut-off age of 14 [10, 17, 24, 27, 41, 43, 45] and 16 years old to classify ASA [25, 32] and thus did not examine the impact of assaults experienced while in college. This is an important limitation as 1.8–34% of college women expereince sexual assault while they are in college [3]. Additionally, college women are at an important development stage for sexual development as a majority of college students are engaging in sexual activity [70]. Thus, it is important to investigate the impact of college sexual assault on sexual functioning of college women.

Additionally, the current review highlighted that all studies investigating the link between sexual violence and sexual functioning among college women are cross-sectional in nature. Further, the relation between sexual violence and sexual functioning has been examined only in 14 unique samples. Thus, there is need for more research with rigorous study designs, including longitudinal designs and experimental designs, in order to increase confidence in findings. Longitudinal research on the relationship between sexual violence and sexual functioning in college women before, during, and after college could begin to explore potential causal relationships among these variables. Meta-analyses that examine for publication bias would help in improving clarity of the results. Further, use of randomized controlled trials of treatments to address sexual functioning following sexual violence could also help to explicate the mechanisms through which sexual violence and poorer sexual functioning are associated. Burgeoning evidence from RCT shows that such cognitive behavioral therapy [71] and psychoeducational training [72] are effective in improving sexual dysfunction and need to adapted and evaluated among survivors of sexual violence. Further, evidence suggests that it is not enough to treat post-trauma distress to improve sexual functioning. For example, a meta-analytic review that included four RCTs found that PTSD treatment did *not* improve sexual functioning in women with histories of sexual violence [73]. The authors concluded that psychological treatment for PTSD has no effect on sexual problems. One of the limitations noted by the meta-analysis was that most interventions did not actively target sexual problems. Thus, future research focused on designing and evaluating sexual functioning intervention for sexual violence survivors is required. Along this vein, the burden of improving sexual functioning should not only be on survivors. Trauma-informed couple-based sexual functioning interventions [74] should be developed and evaluated. Apart from designing interventions focused on sexual functioning, sexual assault prevention programs provided by college student health and education personnel should address potential sexual functioning outcomes following sexual violence. For example, the Enhanced Assess, Acknowledge, Act (EAAA) program utilizes a positive sexuality framework [75]. The program empowers women, including survivors, with skills focused on enhancing positive sexual experiences such as increased awareness of women’s own sexual desire and confidence in asserting in sexual situations.

### Limitations

Findings of the review should be interpreted in the context of limitations. Firstly, one of the inclusion criteria for the systematic review was to include peer-reviewed articles in order to maintain the quality of studies included in the review. Given that there can be publication biases with research, future systematic review should include dissertations as well as non-published studies that may report nonsignificant effects. Secondly, studies on sexual functioning may inadvertently exclude survivors who may choose to abstain from sex. Specifically, measures of sexual function [e.g., Female Sexual Function Index [76]] were developed for women who are sexually active and may fail to take into account the sexual function of survivors histories who abstain from sexual activity. Different profiles of characteristics (e.g., mental health symptoms, assault history) may emerge for survivors who abstain from sexual activity. Thus, future reviews should focus on expanding the definition of sexual functioning to include survivors who are not engaging in sexual activity.

## Conclusions

The current systematic review highlighted the positive link between sexual violence and worsened sexual functioning outcomes among college women. Review demonstrated that the relation between sexual violence and sexual functioning has been examined in only 14 unique samples. Findings suggest a lack of uniformity in definition and measurement of sexual functioning. Results also highlighted the need to examine the association between sexual violence and sexual functioning using longitudinal studies. Post-assault distress such as anxiety and depression contributes to sexual dysfunction among college women survivors. Future studies on community college women, longitudinal studies, and RCTs evaluating interventions for sexual functioning are required.

## Supplementary Information

**Additional file 1.** PRISMA checklist

**Additional file 2.** Search terms used in the review.

## Data Availability

All data on diagnostic yield analyzed during the current study are available in the main text or supplementary material.
