# Supplementary material for: Child sexual abuse, adolescent/adult sexual violence, and sexual functioning among college women: a systematic review
Source: BMC Glob Public Health. 2024 May 13;2:29. doi: 10.1186/s44263-024-00060-7 (PMC11622922; doi:10.1186/s44263-024-00060-7)
Supplement: Supplementary file 2 — Additional file 2. Search terms used in the review. [file 44263_2024_60_MOESM2_ESM.docx]

**Additional File 2**

Full List of Search Terms:

woman or women or female or girl or girls (Topic) and rape* or "sexual assault*" or "sexually assault*" or "sexual violence" or "sexual trauma" or "sexual abuse" or "sexually abused" or "sex victim*" or "sexual victim*" or "partner violence" or "partner sexual violence" or "intimate partner violence" or "intimate partner sexual violence “or molest* (Topic) and arous* or sexuality or orgasm or dysfunction* or satisfied or satisfy* or satisfaction or enjoy* or "sexual well-being" or "sexual self-schema" or function* or "sexual function*" or "sexual adjustment" or "sexual issue*" or "sex therapy" or "sex counseling" or "sexual relationship" or "sexual partner" or pleasure or psychosexual or sex or romantic* or intimacy or intimate or dissatisf* or libido or desire (Topic) not men or male or boy or boys" or alcohol* or "drinking" OR "drug abuse" or "drug use" or "substance abuse" OR cancer or "substance use" OR alcohol* (Topic) and Articles or Review Articles or Early Access or Proceedings Papers (Document Types) and English (Languages)
